# Women’s self-reported symptoms of reproductive tract infection, medical consultations, and factors influencing them in less developed regions: perimenopausal and older women in need of urgent attention

**DOI:** 10.3389/fpubh.2024.1401474

**Published:** 2024-10-22

**Authors:** Ruoyi Zhang, Dan Kang, Siyuan Yang, Dehua Hu, Li Bai, Yongping Ao, Jiaojiao Chen, Yuxian Nie, Xiaowei Zhao, Wei Xu, Qiuling Shi

**Affiliations:** ^1^School of Public Health, Chongqing Medical University, Chongqing, China; ^2^Xian Center for Disease Control and Prevention, Xian, China; ^3^School of Nursing, Chongqing Medical University, Chongqing, China; ^4^Maternal and Child Health, The Maternity Service Centre of Lueyang Maternal and Child Health Care Hospital, Lueyang, China; ^5^State Key Laboratory of Ultrasound in Medicine and Engineering, Chongqing Medical University, Chongqing, China

**Keywords:** reproductive tract infection (RTI), self-reported symptoms, gynecological examinations, women’s health, underdeveloped regions

## Abstract

**Background:**

Reproductive tract infections (RTIs) represent a critical public health concern impacting women’s health, particularly pronounced in developing countries. We aim to investigates the prevalence of self-reported RTI symptoms in women aged 18 to 65 and the factors influencing gynecological examination behavior and associated risks are explored.

**Methods:**

From May 6, 2022, to September 20, 2022, a comprehensive survey engaged 20,864 women aged 18 to 65 in 19 townships in Lueyang, Shaanxi. Each survey team, comprising a gynecologist, two female health center staff, and a master’s student, conducted face-to-face questionnaire surveys in villages and households. The questionnaire featured three sections: prevalence of self-reported RTI symptoms, gynecological examination history, and factors influencing examination behavior.

**Results:**

The high response rate of 98.69% yielded 20,590 valid questionnaires. Among surveyed women, 42.04% reported RTI symptoms, with a higher prevalence in those aged 30 and above, peaking at 44.88% in the 50–59 age group. Vulvar itching (24.73%), abnormal vaginal discharge (17.41%), and urinary tract irritation signs (11.73%) were most common. Older adult women (≥60) reported higher frequency and longer duration of symptoms. Only 9.88% of symptomatic women underwent gynecological examinations in 2022. Examination likelihood decreased with age, presenting a critical gap in healthcare utilization among older women. Reasons for checkups included routine checkups (47.02%), symptom concerns (41.29%), and the availability of free medical checkup programs (9.82%). Barriers included perceived lack of serious symptoms (23.7%), lack of necessity (4.41%) and lack of time (3.98%). Among those examined, 40.58% had a diagnosis of gynecological disorders, including pelvic inflammatory disease, human papilloma virus infection and cervical cancer.

**Conclusion:**

Widespread RTI symptoms, particularly in perimenopausal and older women, underscore the need for enhanced healthcare. Barriers to gynecological examinations include awareness, education, and accessibility issues. Strategies must prioritize health education, routine examinations, and improved healthcare infrastructure in underdeveloped regions.

## 1 Introduction

Reproductive tract infections (RTIs) represent a critical public health concern impacting women’s health, particularly pronounced in developing countries (1). Early prevention of RTI is paramount as they elevate the risk of severe reproductive diseases, including pelvic inflammatory disease, miscarriage, preterm birth, infertility, sexually transmitted infections, and cervical cancer (2). These infections not only inflict reproductive harm upon women of childbearing age but also pose a heightened risk of malignant tumors, such as cervical cancer, among perimenopausal and older adult women (3).

In underdeveloped regions, the prevalence of RTIs may be underestimated due to factors such as lower educational levels, limited autonomy in personal health decisions, lower economic status, inadequate healthcare facilities, insufficient medical resources, and cultural norms of silence concerning diseases (4). These factors contribute to the poor recognition and management of RTIs, leading to a cycle of poor health outcomes and further socio-economic decline. Approximately 38 to 85% of rural women in China are reported to exhibit at least one symptom indicative of RTIs; however, the utilization of healthcare services remains remarkably low (5).

Laboratory testing is considered the gold standard for accurately measuring RTI prevalence. However, the applicability of such tests is limited in rural areas due to insufficient healthcare facilities and a shortage of specialized gynecologists (6). Therefore, in cost-effective terms, self-reporting of RTI symptoms remains a crucial means of assessing the incidence and impact of RTIs, particularly in underdeveloped regions (7). Globally, most cancers (85–90%) are diagnosed on the basis of symptomatic manifestations, despite the availability of cancer screening programs (8), and self-reporting of RTI symptoms contributes to three-early prevention of the disease (9, 10).

Lueyang County in Shaanxi Province is recognized as one of the regions with the highest incidence of cervical cancer and the lowest average income nationwide (11). Therefore, the aim of this study was to investigate the self-reported RTI symptoms, gynecological examination, and related influencing factors of women in Lueyang County, Shaanxi Province, to clarify the relationship between self-reported RTI symptoms and gynecological disorders, and to provide a preventive strategy to improve the rate of gynecological examination and gynecological health of women in underdeveloped areas.

## 2 Methods

### 2.1 Study participants

From May 6, 2022, to September 20, 2022, we conducted a cross-sectional survey of women aged 18 to 65 years in 19 townships in Lueyang County, Shaanxi Province, regarding self-reported RTI symptoms, gynecological examination history, and related influencing factors. The sample for this study was selected based on a census approach, including all eligible women who met the inclusion criteria. The inclusion criteria were: (1) age between 18 and 65 years; (2) residency in Lueyang County for at least 6 months; and (3) complete understanding of the study.

### 2.2 Survey method

Each survey team comprised one gynecologist from Lueyang County Maternal and Child Health Hospital, two female staff members from township health centers, and one master’s degree student from Chongqing Medical University. In total, six survey teams conducted face-to-face questionnaire surveys by visiting villages and households.

### 2.3 Questionnaire design

The survey questionnaire was designed based on a literature review and sought input from professional gynecologists and epidemiologists to ensure its alignment with the specific characteristics of Lueyang County (2, 5, 12–14). The questionnaire consisted of three main sections:

Prevalence of self-reported RTI symptoms in the past 6 months: RTI symptoms included vulvar itching, abnormal vaginal discharge (high volume, yellow color, odor, soybean dregs, bloody, pus, foamy), frequent urination, urgency, painful urination, non-menstrual lower back pain, non-menstrual lower abdominal pain, bleeding after sexual intercourse. Women reporting at least one of these symptoms were considered to have a RTI. The frequency of RTI symptoms was categorized as occasional, sometimes, often, and always. The duration of RTI symptoms was classified as <3 months, 3–6 months, 6 months-1 year, 1–3 years, and > 3 years.

Gynecological examination history and gynecological diagnosis history: whether symptomatic women had a gynecological examination in 2022, reasons for having/not having a gynecological examination, and history of gynecological diagnosis.

Factors influencing women with RTI symptoms who did not undergo gynecological examinations: basic information, number of self-reported RTI symptoms in the past 6 months; menstrual history, marital and reproductive history, sexual behavior, hygiene practices, spouse or sexual partner information.

### 2.4 Statistical analysis

Statistical analyses were conducted using IBM SPSS (version 23.0), SAS (version 9.4), and R (version 4.2.1). Chi-square (χ^2^) tests were employed for the analysis of count data rates (%), revealing differences in the prevalence of various RTI symptoms across different groups. The Cochran-Armitage trend test was utilized to assess the linear relationship between the prevalence of individual RTI symptoms and age. The Jonckheere-Terpstra trend test was applied to evaluate whether there was a linear relationship between the frequency and duration of various RTI symptoms and age.

Univariate and multivariate (Inverse stepwise likelihood ratio test) logistic regression analyses were employed to explore the correlation between women exhibiting RTI symptoms but not undergoing gynecological examinations and the influencing factors obtained from the questionnaire survey. Additionally, the Hosmer-Lemeshow test was conducted to assess the goodness of fit of the models. Risk factors with *p* < 0.1 in the univariate logistic regression analysis were included in the risk regression analysis. The statistical significance level was set at *p* < 0.05.

To address issues related to missing data, the mice package and VIM package in R software were employed to explore the proportion and types of missing data. The pattern of missing data was determined through a correlation matrix called a shadow matrix. Multiple imputation techniques were employed to handle missing data, followed by an assessment of the reliability of the imputation results. After multiple imputation, the interpolation results with the highest Cronbach’s alpha coefficient were used for multifactor analysis.

## 3 Results

### 3.1 Prevalence of self-reported RTI symptoms

A total of 20,864 women were surveyed, and 20,590 valid questionnaires met the inclusion criteria, with a validity rate of 98.69%. A total of 8,657 women (42.04%) reported RTI symptoms in the past 6 months. The prevalence of RTI symptoms exceeded 40% in women aged 30 and above, peaking at 44.88% among those aged 50–59. Individuals with one, two, and multiple (three or more) symptoms constituted 22.93, 11.98, and 7.13% of the total population, respectively (Table 1).

**Table 1:** Prevalence of self-reported symptoms of RTI in the last 6 months among women of different ages.

RTI symptoms in the last six months	Total(%^a^) (*N* = 20,590)	<20(%^b^) (*n* = 142)	20–29(%^b^) (*n* = 1,599)	30–39(%^b^) (*n* = 4,407)	40–49(%^b^) (*n* = 6,289)	50–59(%^b^) (*n* = 6,776)	≥60(%^b^) (*n* = 1,377)	χ^2^^c^	*p*-value^c^	χ^2^^d^	*p*-value^d^
Self-reported symptoms of RTI								285.407	<0.0001	133.6405	<0.0001
Yes	8,657(42.04)	9(6.34)	413(25.83)	1821(41.32)	2,797(44.47)	3,041(44.88)	576(41.83)				
No	11,933(57.96)	133(93.66)	1,186(74.17)	2,586(58.68)	3,492(55.53)	3,735(55.12)	801(58.17)				
Number of symptoms											
1	4,722(22.93)	5(3.52)	234(14.63)	985(22.35)	1,479(23.52)	1709(25.22)	310(22.51)	114.8581	<0.0001	62.4037	<0.0001
2	2,466(11.98)	2(1.41)	109(6.82)	546(12.39)	822(13.07)	820(12.1)	167(12.13)	63.4056	<0.0001	16.3499	<0.0001
≥3	1,469(7.13)	2(1.41)	70(4.37)	290(6.59)	496(7.89)	512(7.56)	99(7.19)	34.6061	<0.0001	17.3647	<0.0001
Types of symptoms											
1. Vulvar itching	5,091(24.73)	5(3.52)	246(15.38)	1,158(26.28)	1,654(26.3)	1702(25.12)	326(23.67)	124.7129	<0.0001	22.4505	<0.0001
2. Abnormal vaginal discharge	3,584(17.41)	4(2.82)	224(14.01)	884(20.06)	1,271(20.21)	1,059(15.63)	142(10.31)	152.9114	<0.0001	19.6728	<0.0001
High volume	1901(9.23)	3(2.11)	116(7.25)	465(10.55)	711(11.31)	532(7.85)	74(5.37)	97.3403	<0.0001	13.2955	0.0003
Yellow color	1,635(7.94)	1(0.7)	92(5.75)	387(8.78)	563(8.95)	526(7.76)	66(4.79)	52.6547	<0.0001	0.6921	0.4054
Odor	1,354(6.58)	1(0.7)	57(3.56)	278(6.31)	510(8.11)	446(6.58)	62(4.5)	65.7908	<0.0001	3.9196	0.0477
Soybean dregs	278(1.35)	0(0)	26(1.63)	104(2.36)	98(1.56)	45(0.66)	5(0.36)	72.6522	<0.0001	45.4977	<0.0001
Bloody	107(0.52)	0(0)	10(0.63)	27(0.61)	39(0.62)	29(0.43)	2(0.15)	7.8885	0.1625	3.7865	0.0517
Pus	102(0.5)	1(0.7)	3(0.19)	34(0.77)	39(0.62)	22(0.32)	3(0.22)	18.1571	0.0028	4.2062	0.0403
Foamy	58(0.28)	0(0)	2(0.13)	13(0.29)	25(0.4)	15(0.22)	3(0.22)	-	0.4012(Fisher)	0.0007	0.9785
3. Frequent urination, urgency, painful urination	2,416(11.73)	1(0.7)	63(3.94)	330(7.49)	731(11.62)	1,059(15.63)	232(16.85)	321.2634	<0.0001	313.9665	<0.0001
4. Non-menstrual lower back pain	1771(8.6)	0(0)	50(3.13)	317(7.19)	539(8.57)	700(10.33)	165(11.98)	131.2436	<0.0001	122.8839	<0.0001
5. Non-menstrual lower abdominal pain	1,681(8.16)	5(3.52)	92(5.75)	356(8.08)	579(9.21)	526(7.76)	123(8.93)	28.174	<0.0001	4.9231	0.0265
6. Bleeding after sexual intercourse	103(0.5)	0(0)	10(0.63)	23(0.52)	48(0.76)	19(0.28)	3(0.22)	18.7832	0.0021	5.3852	0.0203

a Percentage of all women in study.

b Percentage of women in each age group.

c Chi-square test between age groups.

d Cochran-Armitage trend test.

The most common RTI symptoms were vulvar itching (24.73%), abnormal vaginal discharge (17.41%), and urinary symptoms including frequency, urgency, and pain (11.73%). The prevalence of non-menstrual lower back pain (8.6%), non-menstrual lower abdominal pain (8.16%), and post-coital bleeding (0.5%) was comparatively lower. Vulvar itching (26.3%), abnormal vaginal discharge (20.21%), non-menstrual lower abdominal pain (9.21%), and post-coital bleeding (0.76%) were most common in the 40–49 age group. Urinary symptoms (16.85%) and non-menstrual lower back pain (11.98%) showed the highest prevalence in individuals aged 60 and above. The prevalence of most symptoms differed across age groups or showed a linear trend with increasing age.

### 3.2 Frequency of self-reported RTI symptoms

**Figure 1:**
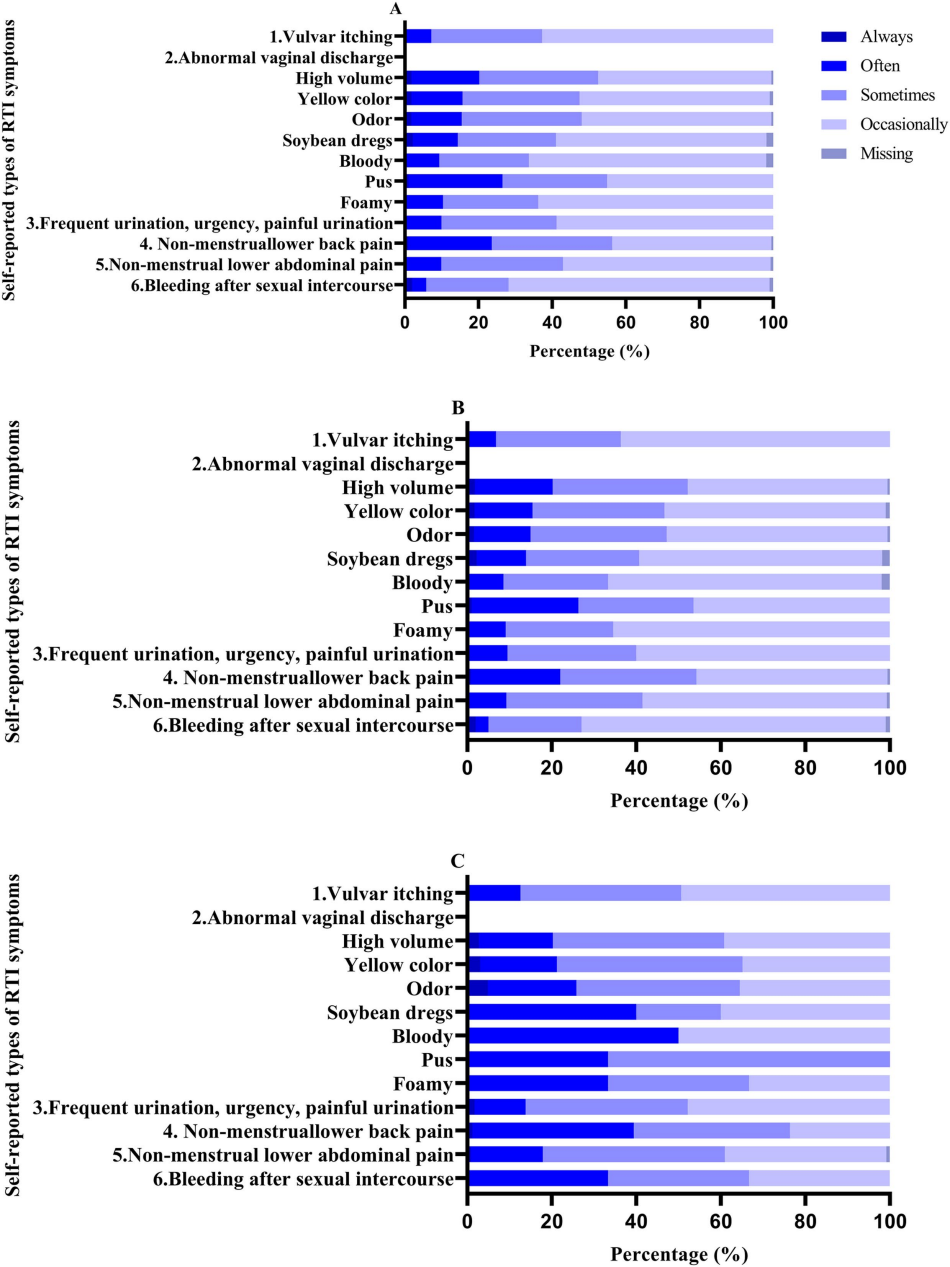
Percentage of self-reported RTI symptom frequencies in patients. A: 18–65 years; b: <60 years; c: ≥60 years.

RTI symptoms in females aged 18–65 predominantly exhibit an occasional occurrence, surpassing 40%. Notably, in women aged 60 and above, the proportion of RTI symptoms occurring occasionally decreases, while the occurrence of these symptoms frequently significantly increases in comparison to women under 60 (Figure 1). The Jonckheere-Terpstra trend test showed that the frequency of vulvar itching, vaginal discharge that was heavy, yellowish in color, and foul smelling, urinary frequency, urgency, and pain of urination, non-menstrual lumbosacral pain, and non-menstrual lower abdominal pain increased with age (*p* < 0.0001; Supplementary Table S1).

### 3.3 Duration of self-reported RTI symptoms

**Figure 2:**
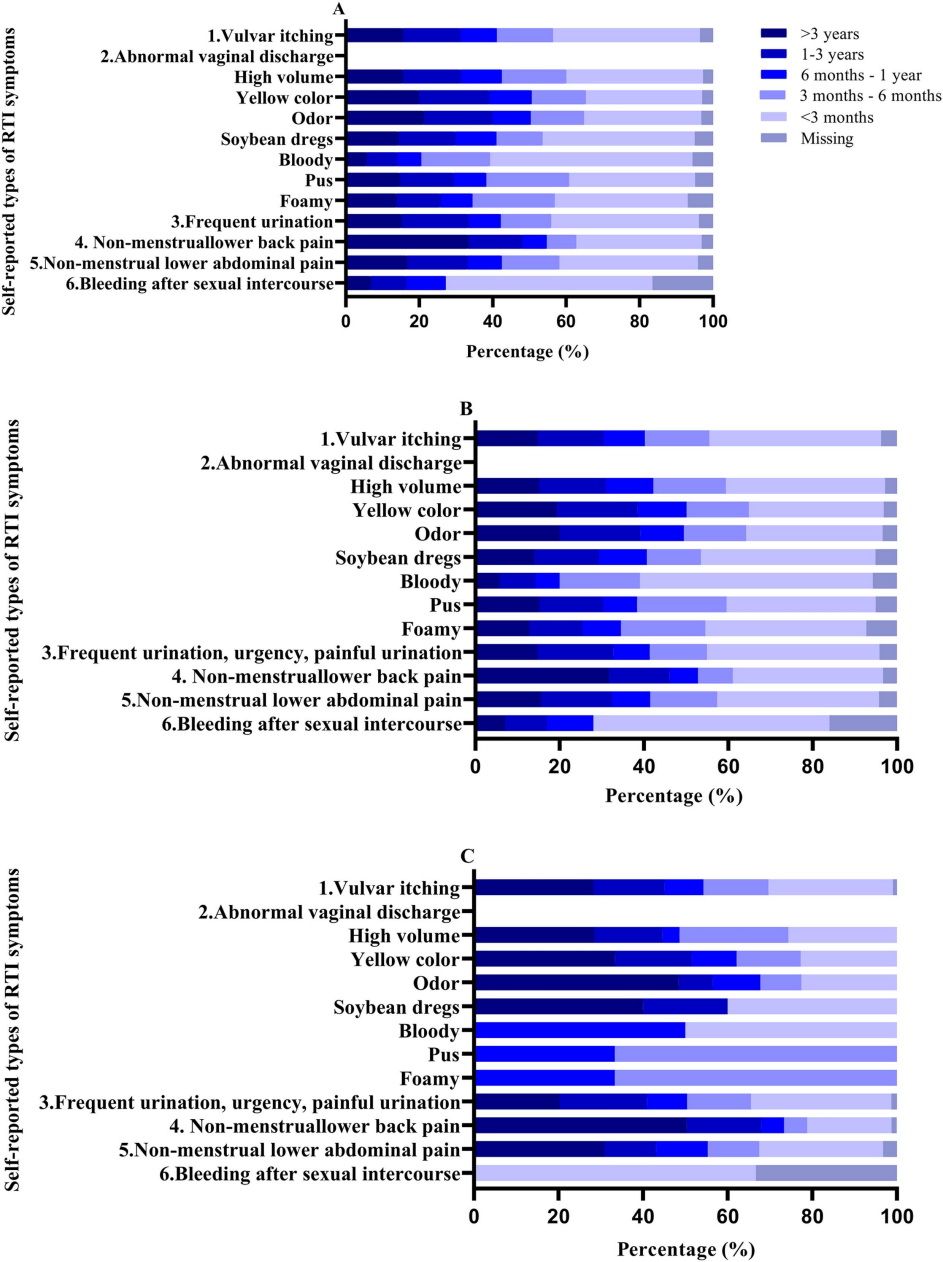
Percentage of duration of self-reported RTI symptoms in patients. A: 18–65 years; b: <60 years; c: ≥60 years.

The majority of individuals experiencing self-reported RTI symptoms reported a duration of less than 3 months (>30%). However, a notable proportion of individuals with non-menstrual lower back pain reported a duration exceeding 3 years (33.37%; Figure 2). In the age group of 60 years and above, non-menstrual lower abdominal pain (30.89%), non-menstrual lower back pain (50.3%), increased vaginal discharge (28.38%), yellow-colored discharge (33.33%), malodorous discharge (48.39%), and discharge resembling soybean dregs (40%) had the highest proportions of durations exceeding 3 years.

According to the Jonckheere-Terpstra trend test, the duration of symptoms for vulvar itching, increased vaginal discharge, yellow-colored discharge, odor, discharge resembling soybean dregs, frequent urination, urgency, painful urination, and non-menstrual lower abdominal pain increased with age (*p* < 0.05; Supplementary Table S2).

### 3.4 Gynecological examination status of patients with self-reported RTI symptoms

Among the 8,657 women who self-reported RTI symptoms in the past 6 months, only 855 individuals (9.88%) underwent gynecological examinations in 2022. Notably, no women under the age of 20 chose to undergo gynecological examinations, only 2.43% of women aged ≥60 years chose to have a gynecological examination. According to the Cochran–Armitage trend test, the likelihood of undergoing gynecological examinations gradually decreased with increasing age (*p* < 0.0001; Table 2). Patients reported that the main reasons for undergoing gynecological examinations in the current year were routine check-ups (47.02%), the presence of symptoms (41.29%), and the availability of free medical check-up programs (9.82%; Table 3).

**Table 2:** Gynecological examination status of patients with self-reported RTI symptoms.

Gynecological examination (Missing = 246)	Total(*N* = 8,657)	<20(*n* = 9)	20–29(*n* = 413)	30–39(*n* = 1821)	40–49(*n* = 2,797)	50–59(*n* = 3,041)	≥60(*n* = 576)	χ^2^^a^	*p*-value^a^	χ^2^^b^	*p*-value^b^
Yes	855(9.88)	0(0)	76(18.4)	269(14.77)	317(11.33)	179(5.89)	14(2.43)	177.0236	<0.0001	168.7479	<0.0001
No	7,556(87.28)	9(100)	328(79.42)	1,513(83.09)	2,406(86.02)	2,755(90.6)	545(94.62)				

a Chi-square test between age groups.

b Cochran-Armitage trend test.

**Table 3:** Reasons for undergoing gynecological examinations among patients with self-reported RTI symptoms (multiple selections allowed).

Reasons for gynecological examinations (Missing = 47)	Total(*N* = 855)	20–29(*n* = 76)	30–39(*n* = 269)	40–49(*n* = 317)	50–59(*n* = 179)	≥60(*n* = 14)
Routine gynecological examination	402(47.02)	37(48.68)	137(50.93)	149(47)	72(40.22)	7(50)
With gynecological symptoms	353(41.29)	24(31.58)	100(37.17)	139(43.85)	87(48.6)	3(21.43)
Free gynecological check-up program	84(9.82)	5(6.58)	24(8.92)	27(8.52)	25(13.97)	3(21.43)
Doctor’s recommendation	52(6.08)	6(7.89)	17(6.32)	17(5.36)	11(6.15)	1(7.14)
Thinking that gynecological examination can solve all gynecological problems	8(0.94)	1(1.32)	2(0.74)	4(1.26)	1(0.56)	0(0)
Pregnancy test/pregnancy preparation	4(0.47)	2(2.63)	2(0.74)	0(0)	0(0)	0(0)
Other reasons	1(0.12)	0(0)	1(0.37)	0(0)	0(0)	0(0)

Patients who did not undergo gynecological examinations in the current year provided various reasons, with the primary factors being the absence of severe symptoms (23.7%), perceived lack of necessity (4.41%), and time constraints (3.98%). Reasons for not undergoing examinations varied across different age groups. For those under 20 years old, the main reason was being young or unmarried (22.22%). Notably, a significant factor for not undergoing examinations in the 20–29 age group (5.79%), 40–49 age group (2.62%), and those aged 60 and above (6.24%) was a lack of awareness about the need for regular gynecological check-ups (Table 4).

**Table 4:** Reasons for not undergoing gynecological examinations among patients with self-reported RTI symptoms (multiple selections allowed).

Reasons for without gynecological examinations (Missing = 5,025)	Total(*N* = 7,556)	<20(*n* = 9)	20–29(*n* = 328)	30–39(*n* = 1,513)	40–49(*n* = 2,406)	50–59(*n* = 2,755)	≥60(*n* = 545)
No serious gynecological symptoms	1791(23.7)	2(22.22)	116(35.37)	361(23.86)	456(18.95)	700(25.41)	156(28.62)
No need	333(4.41)	3(33.33)	20(6.1)	48(3.17)	66(2.74)	161(5.84)	35(6.42)
No time	301(3.98)	1(11.11)	14(4.27)	53(3.5)	63(2.62)	144(5.23)	26(4.77)
Unaware of the need for regular gynecological examinations	264(3.49)	0(0)	19(5.79)	43(2.84)	63(2.62)	105(3.81)	34(6.24)
Do not know the significance	213(2.82)	0(0)	6(1.83)	24(1.59)	52(2.16)	109(3.96)	22(4.04)
Inconvenient transportation	54(0.71)	0(0)	1(0.3)	3(0.2)	8(0.33)	27(0.98)	15(2.75)
Poor economic conditions	38(0.5)	1(11.11)	1(0.3)	4(0.26)	6(0.25)	17(0.62)	9(1.65)
Fear of pain	14(0.19)	1(11.11)	2(0.61)	6(0.4)	2(0.08)	2(0.07)	1(0.18)
Embarrassed	10(0.13)	1(11.11)	2(0.61)	3(0.2)	1(0.04)	1(0.04)	2(0.37)
Inconvenient to move around	4(0.05)	0(0)	0(0)	0(0)	2(0.08)	0(0)	2(0.37)
Young/unmarried	3(0.04)	2(22.22)	0(0)	1(0.07)	0(0)	0(0)	0(0)
Other reasons	30(0.4)	0(0)	3(0.91)	5(0.33)	9(0.37)	11(0.4)	2(0.37)

Out of the 855 symptomatic women who underwent gynecological examinations in 2022, a total of 347 individuals (40.58%) had a documented history of gynecological diagnoses. There were no significant differences in the distribution of gynecological diagnostic history across various age groups (Table 5). The top three diagnosed gynecological conditions were vaginitis (75.22%), cervical erosion (38.04%), and uterine fibroids (30.55%). There were differences in gynecological disorders in various age groups, the important gynecological disorders in 20–29, 30–39, 40–49, and 50–59 years of age were: pelvic inflammatory disease (17.24%), pelvic inflammatory disease (30.10%), cervical cysts (30.94%), and uterine polyps (30.00%), respectively (Table 6).

**Table 5:** Gynecological diagnostic history among patients with self-reported RTI symptoms.

Diagnostic gynecological history (Missing = 8)	Total(*N* = 855)	20–29(*n* = 76)	30–39(*n* = 269)	40–49(*n* = 317)	50–59(*n* = 179)	≥60(*n* = 14)	χ^2^^a^	*p*-value^a^	χ^2^^b^	*p*-value^b^
Yes	347(40.58)	29(38.16)	103(38.29)	139(43.85)	70(39.11)	6(42.86)	2.3773	0.6667	0.3413	0.5591
No	500(58.48)	46(60.53)	164(60.97)	175(55.21)	107(59.78)	8(57.14)				

a Chi-square test between age groups.

b Cochran-Armitage trend test.

**Table 6:** Specific diagnoses of gynecological diseases.

Gynecologic history diagnosis	Total(*N* = 347)	20–29(*n* = 29)	30–39(*n* = 103)	40–49(*n* = 139)	50–59(*n* = 70)	≥60(*n* = 6)
Vaginitis	261(75.22)	19(65.52)	79(76.70)	103(74.10)	55(78.57)	5(83.33)
Cervical erosion	132(38.04)	15(51.72)	62(60.19)	35(25.18)	19(27.14)	1(16.67)
Uterine fibroids	106(30.55)	1(3.45)	18(17.48)	50(35.97)	33(47.14)	4(66.67)
Pelvic inflammatory disease	80(23.05)	5(17.24)	31(30.10)	33(23.74)	11(15.71)	0(0)
Cervical cysts	78(22.48)	1(3.45)	19(18.45)	43(30.94)	15(21.43)	0(0)
Uterine polyps	66(19.02)	1(3.45)	10(9.71)	33(23.74)	21(30.00)	1(16.67)
Cervical polyps	65(18.73)	1(3.45)	10(9.71)	33(23.74)	20(28.57)	1(16.67)
History of ovarian abnormalities	40(11.53)	1(3.45)	14(13.59)	17(12.23)	8(11.43)	0(0)
Inflammation of the cervix	38(10.95)	3(10.34)	15(14.56)	13(9.35)	6(8.57)	1(16.67)
Vulvovaginitis	15(4.32)	1(3.45)	5(4.85)	5(3.6)	4(5.71)	0(0)
Endometriosis	14(4.03)	0(0)	5(4.85)	6(4.32)	3(4.29)	0(0)
Adenomyosis	12(3.46)	0(0)	4(3.88)	6(4.32)	2(2.86)	0(0)
HPV infection	11(3.17)	0(0)	1(0.97)	4(2.88)	5(7.14)	1(16.67)
History of ectopic pregnancy	7(2.02)	0(0)	2(1.94)	2(1.44)	3(4.29)	0(0)
History of tubal abnormalities	5(1.44)	1(3.45)	0(0)	3(2.16)	1(1.43)	0(0)
Pre-cancerous cervical lesions	4(1.15)	0(0)	1(0.97)	2(1.44)	1(1.43)	0(0)
Endometrial cancer	3(0.86)	0(0)	0(0)	0(0)	3(4.29)	0(0)
Cervical cancer	3(0.86)	0(0)	0(0)	0(0)	2(2.86)	1(16.67)
Sexually transmitted diseases	3(0.86)	0(0)	1(0.97)	1(0.72)	0(0)	1(16.67)
Adnexitis	2(0.58)	0(0)	0(0)	1(0.72)	1(1.43)	0(0)
Other gynecologic history	30(8.65)	1(3.45)	9(8.74)	14(13.59)	5(7.14)	1(16.67)

### 3.5 Univariate logistic regression analysis

An exploration of factors influencing the reluctance of patients, reporting symptoms of RTI, to undergo gynecological examinations was conducted through univariate logistic regression analysis. In total, 38 variables were initially subjected to univariate analysis, and subsequently, 23 variables, exhibiting significance at *p* < 0.10, were selected for inclusion in the multivariate analysis (Supplementary Table S3).

### 3.6 Proportion and type of missing data

The missing data type was missing at random. Supplementary Figure S1 delineates the pattern and proportion of missing data for variables included in the multifactorial analysis. Specifically, variables such as household annual income (14.39%) and spouse circumcision history (12.01%) exhibited higher rates of missing data.

### 3.7 Multivariate logistic regression analysis

The multiple imputation for random missing data is detailed in Supplementary Table S4, and the results of the multivariate logistic regression analysis are presented in Table 7. For reference, the analysis without multiple imputation is available in Supplementary Table S5. Women with two or more symptoms, those with two or more marriages, and those with a history of miscarriage showed a lower likelihood of refraining from gynecological examinations after experiencing RTI symptoms. Conversely, factors such as rural residency, infrequent underwear changes (4–7 days per change), low bathing frequency in winter (>7 days/times), and a spouse aged 40 or older were associated with a higher likelihood of non-attendance for gynecological examinations. Moreover, the results from the multivariate logistic regression analysis without multiple imputation indicated that individuals with a BMI of 32 or higher and postmenopausal women were less likely to undergo gynecological examinations.

**Table 7:** Multivariate logistic regression analysis of patients with self-reported RTI symptoms who did not undergo gynecological exams.

Characteristics	Classification	SE	*p*-value	OR[95% CI]
Number of symptoms	–	–	0	–
	1	–	–	1(Reference)
	2	0.085	0.01	0.803[0.679–0.949]
	≥3	0.098	0	0.671[0.554–0.813]
Place of residence(residency >6 months)	–	–	0.032	–
	County	–	–	1(Reference)
	Townships	0.126	0.047	1.285[1.004–1.646]
	Rural	0.096	0.016	1.261[1.044–1.523]
Number of marriages	≤1	–	–	1(Reference)
	≥2	0.163	0.029	0.7[0.508–0.964]
	–	–	0.001	–
Number of miscarriages	0	–	–	1(Reference)
	1–2	0.079	0.002	0.787[0.674–0.919]
	≥3	0.171	0.008	0.634[0.454–0.886]
Menopausal status	menopausal	–	–	1(Reference)
	non-menopausal	0.114	0	0.522[0.417–0.654]
Frequency of changing underwear	–	–	0.01	–
	1–3 days/times	–	–	1(Reference)
	4–7 days/times	0.212	0.003	1.897[1.252–2.875]
	>7 days/times	8653.978	0.998	94081343.43[0-.]
Age of spouse/sexual partner	–	–	0	–
	<30	–	–	1(Reference)
	30–39	0.218	0.11	1.418[0.924–2.176]
	40–49	0.22	0.002	1.974[1.282–3.039]
	50–59	0.226	0.001	2.101[1.35–3.27]
	≥60	0.286	0	3.547[2.025–6.213]
Frequency of bathing in winter	–	–	0.015	–
	1–3 days/times	–	–	1(Reference)
	4–7 days	0.114	0.053	1.247[0.997–1.558]
	8 days-2 weeks/times	0.136	0.005	1.471[1.126–1.922]
	>2 weeks/times	0.169	0.008	1.572[1.128–2.19]

## 4 Discussion

This study reveals the widespread prevalence of self-reported RTI symptoms among women. Women aged 60 and above experience a higher frequency and longer duration of RTI symptoms compared to those under 60. Only a small proportion of women opt for examination when symptomatic. We investigated their reasons for seeking or avoiding gynecological examinations, which are primarily associated with lower levels of awareness, education, economic status, inadequate promotion by healthcare institutions, and difficulties in accessing medical resources.

In this study, the prevalence of self-reported RTI symptoms in women aged 30 and above over the past 6 months exceeds 40%, closely aligning with the national level in India (40%) (15) and surpassing that of rural women in Sichuan Province, China (30.80%) (16) but falling below that of married women in rural Anhui Province, China (58.10%) (17). The most common RTI symptoms in the population are related to urinary tract infections, namely, external genital itching, abnormal vaginal discharge, and urinary irritation, mirroring results from other regions such as India (18, 19). Symptoms linked to cervical cancer, such as non-menstrual lower back pain, non-menstrual lower abdominal pain, and post-coital bleeding, are more prevalent in individuals over 40, emphasizing the need for attention (20). This study suggests that perimenopausal and older women have a higher burden of such symptoms. Women entering menopause and the menopausal period face risks of hormonal changes, gynecological malignancies, and various urogenital system diseases (21). We emphasize the necessity to extend healthcare focus beyond the reproductive period, ensuring perimenopausal and older adult women receive due attention (22, 23).

According to the American College of Obstetricians and Gynecologists (ACOG), all women over the age of 18 are recommended to have annual gynecological exams (24). Similar to other underdeveloped regions, despite the high prevalence of self-reported RTI symptoms among women, the proportion of those seeking gynecological examinations is low (25). Only 2.43% of women over 60 with more severe symptoms choose to undergo gynecological examinations. The primary reasons for older adult women not seeking such examinations are the perception of symptom severity, a lack of necessity, and unawareness of the need for regular gynecological check-ups. Similar reasons have been reported in other studies, where many perceive these symptoms as normal occurrences (26). The fundamental reasons may be attributed to lower levels of awareness and education, insufficient promotion by healthcare institutions, and difficulties in accessing medical resources (4). Conversely, women choosing gynecological examinations report routine check-ups, symptomatic concerns, and access to free medical check-up programs as their main reasons. This suggests that to increase the rate of gynecological examinations among women, efforts should focus on raising awareness and providing free gynecological check-up programs, as demonstrated by China’s initiation of the “two-cancer “free screening program for rural women in 2009, effectively reducing mortality from breast and cervical cancers (27).

In this study, the lower rate of gynecological examinations among symptomatic women with BMI ≥ 32, poor personal hygiene habits, menopausal status, and spouse age ≥ 40 may be attributed to lower levels of education, lower cognitive levels, and poorer health awareness. After we initially identify the reasons why women with RTI symptoms do not undergo gynecological examinations, we can subsequently implement health education measures for these patients to raise awareness, encourage regular gynecological examinations, and promote good hygiene habits. Women residing in rural and communal areas may face lower economic status and unequal distribution of medical resources. Consistent with the results of a review from India (25). Symptomatic women with two or more symptoms, married more than once, or with a history of abortion may be more likely to undergo gynecological examinations, possibly due to the severity of their symptoms. Studies have indicated that health services and delayed treatment are influenced by the severity of symptoms (28).

Among women who underwent gynecological examinations, 40.58% were diagnosed with gynecological diseases, including pelvic inflammatory disease, HPV infection, and cervical cancer, all confirmed to be related to RTIs (29, 30). This indicates that while self-reported symptoms may not be the gold standard for disease diagnosis, it remains a rapid, convenient, and cost-effective choice, aiding in the identification of patients requiring follow-up management, especially in resource-limited regions (5).

The strengths of this study include comprehensive coverage of a large population in Lueyang County using a census approach, which minimizes selection bias. This study also fills a gap in research on RTI symptoms in specific age groups and identifies reasons for delayed gynecological examinations and the actual needs of patients. It provides a scientific basis for improving gynecological health strategies for women in less developed regions. However, this study has limitations. High rates of missing data on household income and sexual behavior were addressed using multiple imputation, highlighting the need for more private and sensitive survey methods in future research. The cross-sectional design also limits the ability to establish causal relationships or track symptom changes over time. Future longitudinal studies are needed to better understand the temporal dynamics of RTI symptoms and their impact on women’s health.

## 5 Conclusion

The study found high self-reported RTI symptoms but limited gynecological examination rates among women in underdeveloped regions. There was a correlation between reported RTI symptoms and gynecological disorders. Targeted health education campaigns focusing on raising awareness of RTI symptoms and the importance of timely gynecological examination, especially for older women and those with lower levels of education, are necessary to improve healthcare utilization in less developed regions. At the same time, health-care providers should strengthen primary health-care infrastructure in rural areas by expanding free gynecological screening programs and increasing the number of trained gynecologists and other necessary medical resources.

## Data Availability

The raw data supporting the conclusions of this article will be made available by the authors, without undue reservation.

## Supplementary material

SUPPLEMENTARY FIGURE S1 Types and proportions of missing data for variables included in the multifactor analysis.
