# Supplementary material for: Women’s self-reported symptoms of reproductive tract infection, medical consultations, and factors influencing them in less developed regions: perimenopausal and older women in need of urgent attention
Source: Front Public Health. 2024 Oct 22;12:1401474. doi: 10.3389/fpubh.2024.1401474 (PMC11534611; doi:10.3389/fpubh.2024.1401474)
Supplement: Supplementary file 2 [file Table_1.DOCX]

**Table S1. Frequency of symptoms in patients with self-reported symptoms of RTI across age group.**

| **Frequency of symptoms** | Total (%^a^) | Occasionally (%^b^) | Sometimes (%^b^) | Often (%^b^) | Always (%^b^) | Z | *P* |
| --- | --- | --- | --- | --- | --- | --- | --- |
| **1**.**Vulvar itching** | | | | | | 11.6076 | **<0.0001** |
| <20 | 5(0.1) | **4(80)** | 1(20) | 0(0) | 0(0) |  |  |
| 20-29 | 246(4.83) | **191(77.64)** | 48(19.51) | 6(2.44) | 1(0.41) |  |  |
| 30-39 | 1158(22.75) | **844(72.88)** | 262(22.63) | 50(4.32) | 2(0.17) |  |  |
| 40-49 | 1654(32.49) | **1034(62.52)** | 518(31.32) | 97(5.86) | 5(0.3) |  |  |
| 50-59 | 1702(33.43) | **960(56.4)** | 579(34.02) | 157(9.22) | 6(0.35) |  |  |
| ≥60 | 326(6.4) | **161(49.39)** | 124(38.04) | 39(11.96) | 2(0.61) |  |  |
| Total | 5091(100) | **3194(62.74)** | 1532(30.09) | 349(6.86) | 16(0.31) |  |  |
| **2**.**Abnormal vaginal discharge** | | | | | | | |
| **High volume (missing=10)** | | | | | | 4.3662 | <0.0001 |
| <20 | 3(0.16) | **2(66.67)** | 0(0) | 1(33.33) | 0(0) |  |  |
| 20-29 | 116(6.1) | **58(50)** | 38(32.76) | 18(15.52) | 2(1.72) |  |  |
| 30-39 | 465(24.46) | **255(54.84)** | 135(29.03) | 66(14.19) | 5(1.08) |  |  |
| 40-49 | 711(37.4) | **328(46.13)** | 226(31.79) | 140(19.69) | 13(1.83) |  |  |
| 50-59 | 532(27.99) | **221(41.54)** | 185(34.77) | 112(21.05) | 12(2.26) |  |  |
| ≥60 | 74(3.89) | 29(39.19) | **30(40.54)** | 13(17.57) | 2(2.7) |  |  |
| Total | 1901(100) | **893(46.98)** | 614(32.3) | 350(18.41) | 34(1.79) |  |  |
| **Yellow color (missing=15)** | | | | | | 4.4949 | **<0.0001** |
| <20 | 1(0.06) | 0(0) | **1(100)** | 0(0) | 0(0) |  |  |
| 20-29 | 92(5.63) | **57(61.96)** | 24(26.09) | 7(7.61) | 2(2.17) |  |  |
| 30-39 | 387(23.67) | **222(57.36)** | 106(27.39) | 49(12.66) | 6(1.55) |  |  |
| 40-49 | 563(34.43) | **293(52.04)** | 189(33.57) | 64(11.37) | 11(1.95) |  |  |
| 50-59 | 526(32.17) | **250(47.53)** | 170(32.32) | 95(18.06) | 8(1.52) |  |  |
| ≥60 | 66(4.04) | 23(34.85) | **29(43.94)** | 12(18.18) | 2(3.03) |  |  |
| Total | 1635(100) | **845(51.68)** | 519(31.74) | 227(13.88) | 29(1.77) |  |  |
| **Odor (missing=7)** | | | | | | 4.581 | **<0.0001** |
| <20 | 1(0.07) | 0(0) | **1(100)** | 0(0) | 0(0) |  |  |
| 20-29 | 57(4.21) | **33(57.89)** | 14(24.56) | 8(14.04) | 1(1.75) |  |  |
| 30-39 | 278(20.53) | **164(58.99)** | 80(28.78) | 30(10.79) | 3(1.08) |  |  |
| 40-49 | 510(37.67) | **273(53.53)** | 169(33.14) | 53(10.39) | 12(2.35) |  |  |
| 50-59 | 446(32.94) | **205(45.96)** | 153(34.3) | 82(18.39) | 4(0.9) |  |  |
| ≥60 | 62(4.58) | 22(35.48) | **24(38.71)** | 13(20.97) | 3(4.84) |  |  |
| Total | 1354(100) | **697(51.48)** | 441(32.57) | 186(13.74) | 23(1.7) |  |  |
| **Soybean dregs (missing=5)** | | | | | | 0.7784 | 0.4363 |
| <20 | 0(0) | 0(.) | 0(.) | 0(.) | 0(.) |  |  |
| 20-29 | 26(9.35) | **16(61.54)** | 6(23.08) | 4(15.38) | 0(0) |  |  |
| 30-39 | 104(37.41) | **59(56.73)** | 31(29.81) | 10(9.62) | 2(1.92) |  |  |
| 40-49 | 98(35.25) | **58(59.18)** | 23(23.47) | 11(11.22) | 3(3.06) |  |  |
| 50-59 | 45(16.19) | **24(53.33)** | 13(28.89) | 7(15.56) | 1(2.22) |  |  |
| ≥60 | 5(1.8) | **2(40)** | 1(20) | **2(40)** | 0(0) |  |  |
| Total | 278(100) | **159(57.19)** | 74(26.62) | 34(12.23) | 6(2.16) |  |  |
| **Bloody (missing=2)** | | | | | | 0.9772 | 0.3285 |
| <20 | 0(0) | 0(.) | 0(.) | 0(.) | 0(0) |  |  |
| 20-29 | 10(9.35) | **7(70)** | 2(20) | 1(10) | 0(0) |  |  |
| 30-39 | 27(25.23) | **19(70.37)** | 7(25.93) | 1(3.7) | 0(0) |  |  |
| 40-49 | 39(36.45) | **24(61.54)** | 12(30.77) | 2(5.13) | 0(0) |  |  |
| 50-59 | 29(27.1) | **18(62.07)** | 5(17.24) | 5(17.24) | 0(0) |  |  |
| ≥60 | 2(1.87) | **1(50)** | 0(0) | **1(50)** | 0(0) |  |  |
| Total | 107(100) | **69(64.49)** | 26(24.3) | 10(9.35) | 0(0) |  |  |
| **Pus** | | | | | | 1.6148 | 0.1063 |
| <20 | 1(0.98) | **1(100)** | 0(0) | 0(0) | 0(0) |  |  |
| 20-29 | 3(2.94) | **3(100)** | 0(0) | 0(0) | 0(0) |  |  |
| 30-39 | 34(33.33) | **16(47.06)** | 11(32.35) | 7(20.59) | 0(0) |  |  |
| 40-49 | 39(38.24) | **16(41.03)** | 11(28.21) | 11(28.21) | 1(2.56) |  |  |
| 50-59 | 22(21.57) | **10(45.45)** | 5(22.73) | 7(31.82) | 0(0) |  |  |
| ≥60 | 3(2.94) | 0(0) | **2(66.67)** | 1(33.33) | 0(0) |  |  |
| Total | 102(100) | **46(45.1)** | 29(28.43) | 26(25.49) | 1(0.98) |  |  |
| **Foamy** | | | | | | 1.007 | 0.3139 |
| <20 | 0(0) | 0(.) | 0(.) | 0(.) | 0(0) |  |  |
| 20-29 | 2(3.45) | **1(50)** | **1(50)** | 0(0) | 0(0) |  |  |
| 30-39 | 13(22.41) | **10(76.92)** | 3(23.08) | 0(0) | 0(0) |  |  |
| 40-49 | 25(43.1) | **15(60)** | 7(28) | 3(12) | 0(0) |  |  |
| 50-59 | 15(25.86) | **10(66.67)** | 3(20) | 2(13.33) | 0(0) |  |  |
| ≥60 | 3(5.17) | **1(33.33)** | **1(33.33)** | **1(33.33)** | 0(0) |  |  |
| Total | 58(100) | **37(63.79)** | 15(25.86) | 6(10.34) | 0(0) |  |  |
| **3**.**Frequent urination, urgency, painful urination** | | | | | | 8.134 | **<0.0001** |
| <20 | 1(0.04) | **1(100)** | 0(0) | 0(0) | 0(0) |  |  |
| 20-29 | 63(2.61) | **52(82.54)** | 9(14.29) | 2(3.17) | 0(0) |  |  |
| 30-39 | 330(13.66) | **235(71.21)** | 80(24.24) | 14(4.24) | 1(0.3) |  |  |
| 40-49 | 731(30.26) | **460(62.93)** | 206(28.18) | 64(8.76) | 1(0.14) |  |  |
| 50-59 | 1059(43.83) | **563(53.16)** | 370(34.94) | 119(11.24) | 7(0.66) |  |  |
| ≥60 | 232(9.6) | **111(47.84)** | 89(38.36) | 28(12.07) | 4(1.72) |  |  |
| Total | 2416(100) | **1422(58.86)** | 754(31.21) | 227(9.4) | 13(0.54) |  |  |
| **4. Non-menstruallower back pain (missing=9)** | | | | | | 7.8727 | **<0.0001** |
| <20 | 0(0) | 0(.) | 0(.) | 0(.) | 0(.) |  |  |
| 20-29 | 50(2.82) | **29(58)** | 14(28) | 7(14) | 0(0) |  |  |
| 30-39 | 317(17.9) | **174(54.89)** | 88(27.76) | 52(16.4) | 1(0.32) |  |  |
| 40-49 | 539(30.43) | **249(46.2)** | 180(33.4) | 100(18.55) | 4(0.74) |  |  |
| 50-59 | 700(39.53) | **274(39.14)** | 236(33.71) | 181(25.86) | 8(1.14) |  |  |
| ≥60 | 165(9.32) | 39(23.64) | 61(36.97) | **63(38.18)** | 2(1.21) |  |  |
| Total | 1771(100) | **765(43.2)** | 579(32.69) | 403(22.76) | 15(0.85) |  |  |
| **5.Non-menstrual lower abdominal pain (missing=12)** | | | | | | 7.4564 | **<0.0001** |
| <20 | 5(0.3) | **3(60)** | 2(40) | 0(0) | 0(0) |  |  |
| 20-29 | 92(5.47) | **71(77.17)** | 17(18.48) | 3(3.26) | 1(1.09) |  |  |
| 30-39 | 356(21.18) | **233(65.45)** | 91(25.56) | 30(8.43) | 0(0) |  |  |
| 40-49 | 579(34.44) | **340(58.72)** | 179(30.92) | 49(8.46) | 4(0.69) |  |  |
| 50-59 | 526(31.29) | **254(48.29)** | 213(40.49) | 55(10.46) | 2(0.38) |  |  |
| ≥60 | 123(7.32) | 47(38.21) | **53(43.09)** | 21(17.07) | 1(0.81) |  |  |
| Total | 1681(100) | **948(56.4)** | 555(33.02) | 158(9.4) | 8(0.48) |  |  |
| **6.Bleeding after sexual intercourse (missing=1)** | | | | | | 0.5763 | 0.5644 |
| <20 | 0(0) | 0(.) | 0(.) | 0(.) | 0(.) |  |  |
| 20-29 | 10(9.71) | **8(80)** | 2(20) | 0(0) | 0(0) |  |  |
| 30-39 | 23(22.33) | **16(69.57)** | 6(26.09) | 0(0) | 0(0) |  |  |
| 40-49 | 48(46.6) | **33(68.75)** | 10(20.83) | 3(6.25) | 2(4.17) |  |  |
| 50-59 | 19(18.45) | **15(78.95)** | 4(21.05) | 0(0) | 0(0) |  |  |
| ≥60 | 3(2.91) | **1(33.33)** | **1(33.33)** | **1(33.33)** | 0(0) |  |  |
| Total | 103(100) | **73(70.87)** | 23(22.33) | 4(3.88) | 2(1.94) |  |  |

^a^ Percentage of each age group in the total number of people with each symptom.

^b^ Frequency of each symptom as a percentage of each age group.

Table S2. Duration of symptoms in self-reported RTI patients across age groups.

| **Duration of Symptoms** | Total | <3 months | 3 months – 6 months | 6 months - 1 year | 1-3 years | >3 years | **Z** | P |
| --- | --- | --- | --- | --- | --- | --- | --- | --- |
| **1.Vulvar itching (missing=184)** | | | | | | | 13.0225 | **<0.0001** |
| <20 | 5(0.1) | **3(60)** | 1(20) | 0(0) | 0(0) | 0(0) |  |  |
| 20-29 | 246(4.83) | **130(52.85)** | 32(13.01) | 28(11.38) | 30(12.2) | 12(4.88) |  |  |
| 30-39 | 1158(22.75) | **541(46.72)** | 219(18.91) | 91(7.86) | 147(12.69) | 88(7.6) |  |  |
| 40-49 | 1654(32.49) | **673(40.69)** | 234(14.15) | 171(10.34) | 270(16.32) | 240(14.51) |  |  |
| 50-59 | 1702(33.43) | **593(34.84)** | 243(14.28) | 180(10.58) | 295(17.33) | 363(21.33) |  |  |
| ≥60 | 326(6.4) | **96(29.45)** | 50(15.34) | 30(9.2) | 55(16.87) | 92(28.22) |  |  |
| Total | 5091(100) | **2036(39.99)** | 779(15.3) | 500(9.82) | 797(15.66) | 795(15.62) |  |  |
| **2.Abnormal vaginal discharge** | | | | | | | | |
| **High volume (missing=52)** | | | | | | | 5.4633 | **<0.0001** |
| <20 | 3(0.16) | **2(66.67)** | 1(33.33) | 0(0) | 0(0) | 0(0) |  |  |
| 20-29 | 116(6.1) | **54(46.55)** | 25(21.55) | 10(8.62) | 11(9.48) | 14(12.07) |  |  |
| 30-39 | 465(24.46) | **188(40.43)** | 93(20) | 54(11.61) | 62(13.33) | 45(9.68) |  |  |
| 40-49 | 711(37.4) | **263(36.99)** | 116(16.32) | 77(10.83) | 128(18) | 111(15.61) |  |  |
| 50-59 | 532(27.99) | **182(34.21)** | 80(15.04) | 66(12.41) | 87(16.35) | 106(19.92) |  |  |
| ≥60 | 74(3.89) | 19(25.68) | 19(25.68) | 3(4.05) | 12(16.22) | **21(28.38)** |  |  |
| Total | 1901(100) | **708(37.24)** | 334(17.57) | 210(11.05) | 300(15.78) | 297(15.62) |  |  |
| **Yellow color (missing=49)** | | | | | | | 7.3779 | **<0.0001** |
| <20 | 1(0.06) | 0(0) | 1(100) | 0(0) | 0(0) | 0(0) |  |  |
| 20-29 | 92(5.63) | **50(54.35)** | 15(16.3) | 8(8.7) | 10(10.87) | 5(5.43) |  |  |
| 30-39 | 387(23.67) | **152(39.28)** | 54(13.95) | 39(10.08) | 72(18.6) | 53(13.7) |  |  |
| 40-49 | 563(34.43) | **165(29.31)** | 91(16.16) | 69(12.26) | 99(17.58) | 118(20.96) |  |  |
| 50-59 | 526(32.17) | **135(25.67)** | 71(13.5) | 66(12.55) | 120(22.81) | 127(24.14) |  |  |
| ≥60 | 66(4.04) | 15(22.73) | 10(15.15) | 7(10.61) | 12(18.18) | **22(33.33)** |  |  |
| Total | 1635(100) | **517(31.62)** | 242(14.8) | 189(11.56) | 313(19.14) | 325(19.88) |  |  |
| **Odor (missing=44)** | | | | | | | 7.2904 | **<0.0001** |
| <20 | 1(0.07) | 0(0) | **1(100)** | 0(0) | 0(0) | 0(0) |  |  |
| 20-29 | 57(4.21) | **29(50.88)** | 5(8.77) | 7(12.28) | 7(12.28) | 6(10.53) |  |  |
| 30-39 | 278(20.53) | **114(41.01)** | 48(17.27) | 25(8.99) | 48(17.27) | 29(10.43) |  |  |
| 40-49 | 510(37.67) | **159(31.18)** | 77(15.1) | 48(9.41) | 102(20) | 105(20.59) |  |  |
| 50-59 | 446(32.94) | 116(26.01) | 60(13.45) | 53(11.88) | 91(20.4) | **118(26.46)** |  |  |
| ≥60 | 62(4.58) | 14(22.58) | 6(9.68) | 7(11.29) | 5(8.06) | **30(48.39)** |  |  |
| Total | 1354(100) | **432(31.91)** | 197(14.55) | 140(10.34) | 253(18.69) | 288(21.27) |  |  |
| **Soybean dregs (missing=14)** | | | | | | | 2.0055 | **0.0449** |
| <20 | 0(0) | 0(.) | 0(.) | 0(.) | 0(.) | 0(.) |  |  |
| 20-29 | 26(9.35) | **11(42.31)** | 5(19.23) | 3(11.54) | 3(11.54) | 3(11.54) |  |  |
| 30-39 | 104(37.41) | **50(48.08)** | 11(10.58) | 12(11.54) | 14(13.46) | 11(10.58) |  |  |
| 40-49 | 98(35.25) | **37(37.76)** | 10(10.2) | 11(11.22) | 15(15.31) | 18(18.37) |  |  |
| 50-59 | 45(16.19) | **15(33.33)** | 9(20) | 5(11.11) | 10(22.22) | 6(13.33) |  |  |
| ≥60 | 5(1.8) | **2(40)** | 0(0) | 0(0) | 1(20) | **2(40)** |  |  |
| Total | 278(100) | **115(41.37)** | 35(12.59) | 31(11.15) | 43(15.47) | 40(14.39) |  |  |
| **Bloody (missing=6)** | | | | | | | -1.8449 | 0.065 |
| <20 | 0(0) | 0(.) | 0(.) | 0(.) | 0(.) | 0(.) |  |  |
| 20-29 | 10(9.35) | **6(60)** | 1(10) | 0(0) | 2(20) | 0(0) |  |  |
| 30-39 | 27(25.23) | **17(62.96)** | 7(25.93) | 0(0) | 1(3.7) | 0(0) |  |  |
| 40-49 | 39(36.45) | **23(58.97)** | 6(15.38) | 4(10.26) | 2(5.13) | 2(5.13) |  |  |
| 50-59 | 29(27.1) | **12(41.38)** | 6(20.69) | 2(6.9) | 4(13.79) | 4(13.79) |  |  |
| ≥60 | 2(1.87) | **1(50)** | 0(0) | **1(50)** | 0(0) | 0(0) |  |  |
| Total | 107(100) | **59(55.14)** | 20(18.69) | 7(6.54) | 9(8.41) | 6(5.61) |  |  |
| **Pus (missing=5)** | | | | | | | 1.0651 | 0.2868 |
| <20 | 1(0.98) | 0(0) | **1(100)** | 0(0) | 0(0) | 0(0) |  |  |
| 20-29 | 3(2.94) | **3(100)** | 0(0) | 0(0) | 0(0) | 0(0) |  |  |
| 30-39 | 34(33.33) | **11(32.35)** | **11(32.35)** | 4(11.76) | 2(5.88) | 4(11.76) |  |  |
| 40-49 | 39(38.24) | **12(30.77)** | 4(10.26) | 4(10.26) | 10(25.64) | 7(17.95) |  |  |
| 50-59 | 22(21.57) | **9(40.91)** | 5(22.73) | 0(0) | 3(13.64) | 4(18.18) |  |  |
| ≥60 | 3(2.94) | 0(0) | **2(66.67)** | 1(33.33) | 0(0) | 0(0) |  |  |
| Total | 102(100) | **35(34.31)** | 23(22.55) | 9(8.82) | 15(14.71) | 15(14.71) |  |  |
| **Foamy (missing=4)** | | | | | | | -0.3239 | 0.746 |
| <20 | 0(0) | 0(.) | 0(.) | 0(.) | 0(.) | 0(.) |  |  |
| 20-29 | 2(3.45) | 0(0) | 0(0) | 0(0) | 0(0) | **1(50)** |  |  |
| 30-39 | 13(22.41) | **6(46.15)** | 0(0) | 1(7.69) | 2(15.38) | 2(15.38) |  |  |
| 40-49 | 25(43.1) | **9(36)** | 6(24) | 2(8) | 3(12) | 4(16) |  |  |
| 50-59 | 15(25.86) | **6(40)** | 5(33.33) | 2(13.33) | 2(13.33) | 0(0) |  |  |
| ≥60 | 3(5.17) | 0(0) | **2(66.67)** | 0(0) | 0(0) | 1(33.33) |  |  |
| Total | 58(100) | **21(36.21)** | 13(22.41) | 5(8.62) | 7(12.07) | 8(13.79) |  |  |
| **3.Frequent urination, urgency, painful urination (missing=94)** | | | | | | | 7.294 | **<0.0001** |
| <20 | 1(0.04) | **1(100)** | 0(0) | 0(0) | 0(0) | 0(0) |  |  |
| 20-29 | 63(2.61) | **37(58.73)** | 9(14.29) | 3(4.76) | 7(11.11) | 4(6.35) |  |  |
| 30-39 | 330(13.66) | **174(52.73)** | 46(13.94) | 28(8.48) | 33(10) | 30(9.09) |  |  |
| 40-49 | 731(30.26) | **302(41.31)** | 98(13.41) | 52(7.11) | 143(19.56) | 100(13.68) |  |  |
| 50-59 | 1059(43.83) | **380(35.88)** | 143(13.5) | 105(9.92) | 211(19.92) | 187(17.66) |  |  |
| ≥60 | 232(9.6) | **77(33.19)** | 35(15.09) | 22(9.48) | 48(20.69) | 47(20.26) |  |  |
| Total | 2416(100) | **971(40.19)** | 331(13.7) | 210(8.69) | 442(18.29) | 368(15.23) |  |  |
| **4.Non-menstrual lower back pain (missing=55)** | | | | | | | 0.4373 | 0.6619 |
| <20 | 0(0) | 0(.) | 0(.) | 0(.) | 0(.) | 0(.) |  |  |
| 20-29 | 50(2.82) | **25(50)** | 6(12) | 7(14) | 7(14) | 5(10) |  |  |
| 30-39 | 317(17.9) | **141(44.48)** | 28(8.83) | 25(7.89) | 48(15.14) | 55(17.35) |  |  |
| 40-49 | 539(30.43) | **210(38.96)** | 47(8.72) | 41(7.61) | 80(14.84) | 139(25.79) |  |  |
| 50-59 | 700(39.53) | 196(28) | 52(7.43) | 36(5.14) | 96(13.71) | **309(44.14)** |  |  |
| ≥60 | 165(9.32) | 33(20) | 9(5.45) | 9(5.45) | 29(17.58) | **83(50.3)** |  |  |
| Total | 1771(100) | **605(34.16)** | 142(8.02) | 118(6.66) | 260(14.68) | **591(33.37)** |  |  |
| **5.Non-menstrual lower abdominal pain (missing=70)** | | | | | | | 6.178 | **<0.0001** |
| <20 | 5(0.3) | **3(60)** | 0(0) | 0(0) | 0(0) | 0(0) |  |  |
| 20-29 | 92(5.47) | **44(47.83)** | 16(17.39) | 11(11.96) | 9(9.78) | 7(7.61) |  |  |
| 30-39 | 356(21.18) | **150(42.13)** | 60(16.85) | 28(7.87) | 56(15.73) | 40(11.24) |  |  |
| 40-49 | 579(34.44) | **227(39.21)** | 90(15.54) | 48(8.29) | 107(18.48) | 81(13.99) |  |  |
| 50-59 | 526(31.29) | **174(33.08)** | 82(15.59) | 54(10.27) | 91(17.3) | 114(21.67) |  |  |
| ≥60 | 123(7.32) | 36(29.27) | 15(12.2) | 15(12.2) | 15(12.2) | **38(30.89)** |  |  |
| Total | 1681(100) | **634(37.72)** | 263(15.65) | 156(9.28) | 278(16.54) | 280(16.66) |  |  |
| **6.Bleeding after sexual intercourse (missing=17)** | | | | | | | 0.2869 | 0.7742 |
| <20 | 0(0) | 0(.) | 0(0) | 0(.) | 0(.) | 0(.) |  |  |
| 20-29 | 10(9.71) | **8(80)** | 0(0) | 1(10) | 0(0) | 0(0) |  |  |
| 30-39 | 23(22.33) | **12(52.17)** | 0(0) | 4(17.39) | 1(4.35) | 1(4.35) |  |  |
| 40-49 | 48(46.6) | **22(45.83)** | 0(0) | 5(10.42) | 7(14.58) | 5(10.42) |  |  |
| 50-59 | 19(18.45) | **14(73.68)** | 0(0) | 1(5.26) | 2(10.53) | 1(5.26) |  |  |
| ≥60 | 3(2.91) | **2(66.67)** | 0(0) | 0(0) | 0(0) | 0(0) |  |  |
| Total | 103(100) | **58(56.31)** | 0(0) | 11(10.68) | 10(9.71) | 7(6.8) |  |  |

^a^ Percentage of each age group in the total number of people with each symptom.

^b^ Duration of each symptom as a percentage of each age group.

Table S3. Univariate logistic regression analysis of failure to undergo gynecologic examination in patients with self-reported symptoms of RTI.

| Characteristics |  | Women with RTI symptoms  N(%^a^) | Women without gynecological examination n(%^b^) | Women with gynecological examination  (%^b^) | χ^2^ | *P*-Value |
| --- | --- | --- | --- | --- | --- | --- |
| Age | <20 | 9(0.11) | 9(100) | 0(0) | 177.0236 | **<0.0001** |
|  | 20-29 | 404(4.8) | 328(81.19) | 76(18.81) |  |  |
|  | 30-39 | 1782(21.19) | 1513(84.9) | 269(15.1) |  |  |
|  | 40-49 | 2723(32.37) | 2406(88.36) | 317(11.64) |  |  |
|  | 50-59 | 2934(34.88) | 2755(93.9) | 179(6.1) |  |  |
|  | ≥60 | 559(6.65) | 545(97.5) | 14(2.5) |  |  |
| BMI | ＜18.5 | 318(3.78) | 281(88.36) | 37(11.64) | 8.4262 | **0.0772** |
|  | 18.5≤BMI<24 | 4432(52.69) | 3976(89.71) | 456(10.29) |  |  |
|  | 24≤BMI<28 | 2798(33.27) | 2504(89.49) | 294(10.51) |  |  |
|  | 28≤BMI<32 | 724(8.61) | 662(91.44) | 62(8.56) |  |  |
|  | ≥32 | 139(1.65) | 133(95.68) | 6(4.32) |  |  |
| Educational level | Illiteracy/no formal education | 1421(16.94) | 1341(94.37) | 80(5.63) | 109.34 | **<0.0001** |
|  | Elementary school and below | 2928(34.9) | 2676(91.39) | 252(8.61) |  |  |
|  | Junior high school | 2194(26.15) | 1968(89.7) | 226(10.3) |  |  |
|  | High school and above | 1846(22.01) | 1550(83.97) | 296(16.03) |  |  |
| Place of residence  (residency > 6 months) | County | 2303(27.42) | 1980(85.97) | 323(14.03) | 54.4347 | **<0.0001** |
|  | Townships | 1014(12.07) | 911(89.84) | 103(10.16) |  |  |
|  | Rural | 5081(60.5) | 4653(91.58) | 428(8.42) |  |  |
| Ethnic group | Han | 8340(99.29) | 7492(89.83) | 848(10.17) | - | 0.3831 |
|  | Hui | 47(0.56) | 45(95.74) | 2(4.26) |  |  |
|  | Others | 13(0.15) | 12(92.31) | 1(7.69) |  |  |
| Religious beliefs | No | 8312(98.94) | 7467(89.83) | 845(10.17) | 0.0003 | 0.9867 |
|  | Yes | 89(1.06) | 80(89.89) | 9(10.11) |  |  |
| Household annual income | ＜5000 CYN | 224(3.11) | 205(91.52) | 19(8.48) | 25.4962 | **<0.0001** |
|  | 5000-10000 CYN | 800(11.11) | 728(91) | 72(9) |  |  |
|  | 10000-30000 CYN | 3357(46.62) | 3060(91.15) | 297(8.85) |  |  |
|  | 30000-100000 CYN | 2506(34.8) | 2215(88.39) | 291(11.61) |  |  |
|  | >100000 CYN | 314(4.36) | 264(84.08) | 50(15.92) |  |  |
| Smoking history | Never | 8242(98.11) | 7400(89.78) | 842(10.22) | 2.6392 | 0.1043 |
|  | Smoke or quit smoking | 159(1.89) | 149(93.71) | 10(6.29) |  |  |
| Alcohol consumption history | Never | 7698(91.62) | 6942(90.18) | 756(9.82) | 10.3057 | **0.0013** |
|  | Drinking or abstaining from alcohol | 704(8.38) | 608(86.36) | 96(13.64) |  |  |
| Number of self-reported RTI symptoms | 1 | 4602(54.71) | 4187(90.98) | 415(9.02) | 17.582 | **0.0002** |
|  | 2 | 2393(28.45) | 2132(89.09) | 261(10.91) |  |  |
|  | ≥3 | 1416(16.84) | 1237(87.36) | 179(12.64) |  |  |
| Age at menarche | ＜12 | 185(2.22) | 161(87.03) | 24(12.97) | 2.2823 | 0.3195 |
|  | 12-18 | 8111(97.23) | 7290(89.88) | 821(10.12) |  |  |
|  | >18 | 46(0.55) | 43(93.48) | 3(6.52) |  |  |
| Menopausal status | Menopausal | 3063(36.62) | 2917(95.23) | 146(4.77) | 152.5034 | **<0.0001** |
|  | Non-menopausal | 5302(63.38) | 4601(86.78) | 701(13.22) |  |  |
| Age at menopause | ＜45 | 329(10.77) | 314(95.44) | 15(4.56) | 0.192 | 0.9084 |
|  | 45-55 | 2656(86.97) | 2529(95.22) | 127(4.78) |  |  |
|  | >55 | 69(2.26) | 65(94.2) | 4(5.8) |  |  |
| Regularity of menstrual cycles in the past six months | Regular | 3603(68.26) | 3122(86.65) | 481(13.35) | 0.6577 | 0.7198 |
|  | Irregular | 1137(21.54) | 987(86.81) | 150(13.19) |  |  |
|  | Uncertain  (in perimenopause) | 538(10.19) | 473(87.92) | 65(12.08) |  |  |
| Dysmenorrhea in the past six months | No | 2839(54.04) | 2477(87.25) | 362(12.75) | 1.2246 | 0.2685 |
|  | Yes | 2415(45.96) | 2082(86.21) | 333(13.79) |  |  |
| Marital status | Married | 7941(94.59) | 7131(89.8) | 810(10.2) | 14.7416 | **0.0021** |
|  | Divorced | 125(1.49) | 103(82.4) | 22(17.6) |  |  |
|  | Widowed | 215(2.56) | 205(95.35) | 10(4.65) |  |  |
|  | Unmarried | 114(1.36) | 102(89.47) | 12(10.53) |  |  |
| Number of marriages | ≤1 | 8042(95.96) | 7240(90.03) | 802(9.97) | 6.2563 | **0.0124** |
|  | ≥2 | 339(4.04) | 291(85.84) | 48(14.16) |  |  |
| Age at first marriage | <18 | 70(0.85) | 63(90) | 7(10) | 0.0109 | 0.9946 |
|  | 18-30 | 8128(98.46) | 7304(89.86) | 824(10.14) |  |  |
|  | >30 | 57(0.69) | 51(89.47) | 6(10.53) |  |  |
| Age at first childbirth | <18 | 32(0.39) | 29(90.63) | 3(9.38) | 1.5262 | 0.4662 |
|  | 18-30 | 7910(97.47) | 7128(90.11) | 782(9.89) |  |  |
|  | >30 | 173(2.13) | 151(87.28) | 22(12.72) |  |  |
| Number of pregnancies | 0 | 104(1.24) | 86(82.69) | 18(17.31) | 13.2879 | **0.0013** |
|  | 1-2 | 5391(64.42) | 4885(90.61) | 506(9.39) |  |  |
|  | ≥3 | 2874(34.34) | 2550(88.73) | 324(11.27) |  |  |
| Number of childbirths | 0 | 189(2.25) | 155(82.01) | 34(17.99) | 21.5124 | **<0.0001** |
|  | 1-2 | 7636(91.03) | 6857(89.8) | 779(10.2) |  |  |
|  | ≥3 | 563(6.71) | 527(93.61) | 36(6.39) |  |  |
| Number of miscarriages | 0 | 5404(64.53) | 4939(91.4) | 465(8.6) | 39.388 | **<0.0001** |
|  | 1-2 | 2662(31.79) | 2329(87.49) | 333(12.51) |  |  |
|  | ≥3 | 308(3.68) | 261(84.74) | 47(15.26) |  |  |
| History of first sexual intercourse | Yes | 8272(99.18) | 7432(89.85) | 840(10.15) | 0.1914 | 0.6618 |
|  | No | 68(0.82) | 60(88.24) | 8(11.76) |  |  |
| Age at first sexual intercourse | <18 | 91(1.1) | 82(90.11) | 9(9.89) | 0.0693 | 0.9659 |
|  | 18-30 | 8146(98.48) | 7319(89.85) | 827(10.15) |  |  |
|  | >30 | 35(0.42) | 31(88.57) | 4(11.43) |  |  |
| Sexual intercourse in the last year | Yes | 5285(67.08) | 4670(88.36) | 615(11.64) | 46.9101 | **<0.0001** |
|  | No | 2594(32.92) | 2420(93.29) | 174(6.71) |  |  |
| Most commonly used menstrual products | Tampons | 5106(60.79) | 4472(87.58) | 634(12.42) | - | **<0.0001** |
|  | Tampons | 18(0.21) | 13(72.22) | 5(27.78) |  |  |
|  | Tissue paper | 3210(38.22) | 2999(93.43) | 211(6.57) |  |  |
|  | Other paper | 38(0.45) | 35(92.11) | 3(7.89) |  |  |
|  | Cloth | 25(0.3) | 25(100) | 0(0) |  |  |
|  | Others | 2(0.02) | 2(100) | 0(0) |  |  |
| Most commonly used materials for toilets | Tissue paper | 8372(99.77) | 7518(89.8) | 854(10.2) | - | 0.714 |
|  | Others | 19(0.23) | 18(94.74) | 1(5.26) |  |  |
| Frequency of bathing in summer | 1-3 days/times | 6704(79.92) | 5961(88.92) | 743(11.08) | 30.287 | **<0.0001** |
|  | 4-7 days/times | 1401(16.7) | 1308(93.36) | 93(6.64) |  |  |
|  | 8 days-2 weeks/times | 197(2.35) | 181(91.88) | 16(8.12) |  |  |
|  | >2 weeks/times | 86(1.03) | 83(96.51) | 3(3.49) |  |  |
| Frequency of bathing in winter | 1-3 days/times | 785(9.39) | 660(84.08) | 125(15.92) | 58.6808 | **<0.0001** |
|  | 4-7 days/times | 4558(54.5) | 4059(89.05) | 499(10.95) |  |  |
|  | 8 days-2 weeks/times | 1891(22.61) | 1735(91.75) | 156(8.25) |  |  |
|  | >2 weeks/times | 1130(13.51) | 1060(93.81) | 70(6.19) |  |  |
| Frequency of changing underwear | 1-3 days/times | 7746(92.38) | 6917(89.3) | 829(10.7) | 30.1144 | **<0.0001** |
|  | 4-7 days/times | 618(7.37) | 593(95.95) | 25(4.05) |  |  |
|  | >7days/times | 21(0.25) | 21(100) | 0(0) |  |  |
| Bath before intercourse | No bathing | 362(7.12) | 333(91.99) | 29(8.01) | 10.1303 | **0.0063** |
|  | Occasionally | 2011(39.54) | 1795(89.26) | 216(10.74) |  |  |
|  | Always | 2713(53.34) | 2364(87.14) | 349(12.86) |  |  |
| Bath after intercourse | No bathing | 482(9.48) | 439(91.08) | 43(8.92) | 11.5213 | **0.0031** |
|  | Occasionally | 2079(40.89) | 1861(89.51) | 218(10.49) |  |  |
|  | Always | 2523(49.63) | 2192(86.88) | 331(13.12) |  |  |
| Spouse or sexual partner information | | | | | | |
| Presence or absence of spouse or sexual partner | Yes | 7750(94.19) | 6954(89.73) | 796(10.27) | 1.7858 | 0.1814 |
|  | No | 478(5.81) | 438(91.63) | 40(8.37) |  |  |
| Age | <30 | 139(1.82) | 107(76.98) | 32(23.02) | 161.2299 | **<0.0001** |
|  | 30-39 | 1239(16.26) | 1027(82.89) | 212(17.11) |  |  |
|  | 40-49 | 2186(28.68) | 1927(88.15) | 259(11.85) |  |  |
|  | 50-59 | 3075(40.35) | 2831(92.07) | 244(7.93) |  |  |
|  | ≥60 | 982(12.89) | 948(96.54) | 34(3.46) |  |  |
| Educational level | Illiteracy/no formal education | 531(6.88) | 499(93.97) | 32(6.03) | 61.3641 | **<0.0001** |
|  | Elementary school and below | 2611(33.82) | 2415(92.49) | 196(7.51) |  |  |
|  | Junior high school | 2601(33.69) | 2309(88.77) | 292(11.23) |  |  |
|  | High school and above | 1978(25.62) | 1705(86.2) | 273(13.8) |  |  |
| Circumcision history | Yes | 196(2.83) | 165(84.18) | 31(15.82) | 6.6465 | **0.0099** |
|  | No | 6727(97.17) | 6045(89.86) | 682(10.14) |  |  |
| Smoking history | No | 2657(34.37) | 2393(90.06) | 264(9.94) | 0.5332 | 0.4653 |
|  | Smoke or quit smoking | 5073(65.63) | 4542(89.53) | 531(10.47) |  |  |
| Alcohol consumption history | Never | 2747(35.55) | 2485(90.46) | 262(9.54) | 2.2589 | 0.1328 |
|  | Drinking alcohol or have quit | 4981(64.45) | 4452(89.38) | 529(10.62) |  |  |

Table S4. Multiple interpolation methods.

| Seed | Number of imputations | Cronbach alpha coefficient | χ2  (Hosmer-Lemeshow test) | *P*-Value | Correct prediction rate(%) |
| --- | --- | --- | --- | --- | --- |
| 20 000 000 | 5 | 0.413 | 7.965 | 0.437 | 89.8 |

**Table S5. Multivariate logistic regression analysis of patients with self-reported RTI symptoms who did not undergo gynecological exams (without multiple interpolation).**

| Characteristics | classification | SE | *P*-Value | OR(95% CI) |
| --- | --- | --- | --- | --- |
| Number of symptoms | - | - | 0 | - |
|  | 1 | - | - | 1(Reference) |
|  | 2 | 0.102 | 0.002 | 0.729[0.597-0.891] |
|  | ≥3 | 0.119 | 0 | 0.597[0.473-0.754] |
| BMI | - | - | 0.034 | - |
|  | ＜18.5 | - | - | 1(Reference) |
|  | 18.5≤BMI<24 | 0.229 | 0.352 | 1.238[0.79-1.941] |
|  | 24≤BMI<28 | 0.234 | 0.981 | 1.006[0.636-1.591] |
|  | 28≤BMI<32 | 0.275 | 0.239 | 1.383[0.806-2.372] |
|  | ≥32 | 0.63 | 0.033 | 3.838[1.116-13.196] |
| Place of residence  (Residency > 6 months) | - | - | 0.001 | - |
|  | County | - | - | 1(Reference) |
|  | Townships | 0.151 | 0.013 | 1.458[1.084-1.962] |
|  | Rural | 0.101 | 0.001 | 1.419[1.163-1.731] |
| Number of marriages | ≤1 | - | - | 1(Reference) |
|  | ≥2 | 0.189 | 0.013 | 0.626[0.432-0.908] |
| Number of miscarriages | - | - | 0.011 | - |
|  | 0 | - | - | 1(Reference) |
|  | 1-2 | 0.095 | 0.025 | 0.808[0.671-0.974] |
|  | ≥3 | 0.197 | 0.014 | 0.615[0.418-0.905] |
| Menopausal status | Menopausal | - | - | 1(Reference) |
|  | Non-menopausal | 0.161 | 0.001 | 0.599[0.437-0.82] |
| Frequency of changing underwear | - | - | 0.008 | - |
|  | 1-3 days/times | - | - | 1(Reference) |
|  | 4-7 days/times | 0.318 | 0.002 | 2.691[1.444-5.017] |
|  | >7 days/times | 10972.707 | 0.999 | 36958660.34[0-.] |
